# Decoding Hip Muscle Activation: A Comparative Electromyographic Analysis of Turn-Out Bent Knee Pulse and Single-Leg Banded Glute Bridge Exercises in Healthy Female Subjects

**DOI:** 10.3390/ejihpe13090117

**Published:** 2023-08-26

**Authors:** Qais Gasibat, Cristina Ioana Alexe, Gabriela Raveica, Dragoș Ioan Tohănean, Koronas Vasilios, Dan Iulian Alexe

**Affiliations:** 1 Department of Sports Studies, University Putra Malaysia UPM, Selangor 43400, Malaysia; gs57022@student.upm.edu.my; 2 Department of Physical Education and Sport Performance, “Vasile Alecsandri” University of Bacău, 600115 Bacău, Romania; 3 Department of Physical and Occupational Therapy, “Vasile Alecsandri” University of Bacău, 600115 Bacău, Romania; gabriela.raveica@ub.ro; 4 Department of Motric Performance, “Transilvania” University of Brașov, 600115 Brașov, Romania; dragos.tohanean@unitbv.ro; 5 Department Private College Apostolas Pavlos, 55535 Thessaloniki, Greece; b.koronas@yahoo.gr

**Keywords:** electromyography, core stability, gluteus muscles, exercises

## Abstract

Individuals with lower extremity injuries and back pain may exhibit weakness and stiffness in important muscles such as the gluteus maximus and external hip rotators. To aid clinicians in understanding the impact of exercises on factors like stability, endurance, and strength, electromyography (EMG) examination can be employed to monitor muscle activation. In this investigation, the EMG activity of the gluteus maximus and medius were compared between two exercises: the turn-out bent knee pulse and the single-leg banded glute bridge. The study enrolled a group of 64 healthy young women, aged 19 to 24 years. The raw data collected were standardized and represented as a percentage of maximum voluntary isometric contraction (%MVIC). To assess the reliability of the EMG recordings, the test–retest analysis was performed using the intraclass correlation coefficient (ICC3,1). Statistical analysis involved conducting a one-way ANOVA to compare the EMG amplitudes between the two exercises. Remarkably, the results demonstrated a significantly higher EMG signal amplitude during the single-leg banded glute bridge exercise (mean ± SD: 90 ± 28% MVIC) when compared to the turn-out bent knee pulse exercise (mean ± SD: 70 ± 15% MVIC) (F = 16.584, *p* = 0.001). The study found that the single-leg banded glute bridge exercise had a significantly higher EMG signal amplitude compared to the turn-out bent knee pulse exercise. This suggests that the single-leg banded glute bridge exercise may be more effective in strengthening the gluteus maximus and medius muscles. Overall, this study highlights the importance of targeted muscle training in rehabilitation protocols and the use of EMG examination to monitor muscle activation.

## 1. Introduction

In recent studies, researchers have highlighted the significance of targeted training for the hip and thigh muscles to prevent sports-related injuries [1,2]. Significantly, individuals with lower extremity injuries and back pain often exhibit weakness and limited endurance in crucial muscles such as the gluteus maximus and external hip rotators [3,4,5]. Notably, Leetun et al. [5] made the observation that athletes who were able to avoid injuries had considerably stronger hip abductor and external rotator muscles. Furthermore, studies have reported that females experiencing patellofemoral joint pain tend to exhibit weaker hip abductors, extensors, and external rotators compared to their counterparts of the same age [6,7]. In a compelling case series involving two patients, Mascal et al. [8] demonstrated significant improvements in patellofemoral pain, lower extremity movements, and overall functionality by targeting the strengthening of these muscles. These findings can be considered real arguments for the importance of specific muscle training in preventing injury and facilitating recovery.

Within the literature, there has been ongoing speculation about the relevance of the gluteus medius in relation to low back pain, despite its clear functional connection to the spinopelvic system [9]. In theory, all three muscles, with their unique anatomical structures and associated lines of action, play a vital role in stabilizing the hip, preventing hip misalignment, and maintaining pelvic stability during a single-leg stance and walking, both in healthy individuals and injured patients [10]. Significantly, prolonged sitting can lead to a shortening and tightening of the front hip muscles (particularly the iliopsoas, or hip flexors), while the gluteal muscles in the back become elongated and weak [11]. Surprisingly, the body tends to forget how to activate the gluteal muscles, redirecting neural signals intended for them to nearby muscles in order to compensate for their weakness [11]. Furthermore, there is a strong association between lumbar pain and the muscles surrounding the hips, as the muscles in the waist region have a significant positive impact on low back pain. Specifically, the strength of the hip extensors and the endurance of the hip adductors may contribute to lumbar pain [12,13]. Additionally, hip abductor weakness is a common finding among individuals with low back pain [14]. People who experience low back pain often exhibit reduced muscle strength, endurance, and flexibility, as well as limitations in lumbar and lower limb joint mobility [12,15]. The intricate relationship between these factors highlights the complex nature of low back pain and its connection to hip muscles and mobility [9,10].

When individuals experience low back pain, they often tend to avoid movements that cause pain, leading to reduced activity and resulting in weakened gluteus maximus muscles due to disuse [9]. Notably, research has found that people with low back pain experience more rapid fatigue in their gluteus maximus muscles [16]. Furthermore, avoiding movements that aggravate the degenerative spine can lead to the deconditioning of the back and hip extensor muscles [17]. The discovery of increased fatigue levels in the gluteus maximus highlights the importance of incorporating this muscle into low back pain rehabilitation in order to break the cycle of pain avoidance behavior [17,18]. Moreover, apart from its role in injury prevention, studies have demonstrated that targeted strengthening of the core hip muscles can enhance athletic performance [19]. The hip muscles, including the gluteus muscles, have been shown to be crucial in generating maximum power and precision during activities such as golf and baseball swings [20]. Therefore, athletes who want to improve their overall performance in these movements can benefit from focusing on strengthening these muscles.

In line with understanding the role of hip muscles, groundbreaking investigations conducted by Ekstrom et al. [21] and Qais et al. [17] have provided comprehensive data on the electromyography (EMG) activity of the gluteus muscles during various lower back-related activities. These studies have contributed to our understanding of how the gluteus muscles are involved in different movements and exercises. However, despite researchers concentrating on EMG studies during specific exercises [22], there is currently no conclusive evidence favoring one exercise program over another for effectively engaging the gluteus muscles during the rehabilitation of individuals with low back pain.

As a result, a diverse array of exercises have been implemented to gradually enhance the strength of the gluteus muscles, taking into consideration the specific needs and limitations of individuals with low back pain [17,21,22]. This approach recognizes the importance of tailored rehabilitation programs that target the gluteus muscles to promote strengthening and improve overall function.

Significantly, certain exercises, like the turn-out bent knee pulse and single-leg banded glute bridge exercise, have not yet undergone investigation using EMG analysis to assess their effectiveness in activating gluteus muscles and reducing low back pain in females. EMG serves as the gold standard measurement tool for examining muscle activity, enabling therapists to analyze the levels of hip muscle activation during training in order to select the most suitable exercises. Therefore, the primary objective of this study is to investigate the EMG activity of the gluteus muscles during the turn-out bent knee pulse exercise and compare it with the single-leg banded glute bridge exercise in healthy female participants. Our hypothesis posits that specific exercises will yield significantly higher levels of EMG signal amplitude in the analyzed muscles compared to other exercises.

## 2. Materials and Methods

### 2.1. Participants

A prospective, single-group, repeated-measures design was utilized in this study to examine the activation level of the gluteus maximus and medius muscles during two exercises employed in lumbar spine rehabilitation. In order to determine the appropriate sample size for this study, G*Power software (version 3.1.6) was employed, with an alpha level of 0.05 and an effect size of 0.23 [17]. For the calculation of EMG findings for the gluteus maximus and medius muscles, the sample includes 24 participants, with results in one arm only. However, to avoid drop out among participants, a total of 64 young females (healthy, untrained), aged between 19 and 24 years, with a BMI ranging from 18.5 to 24.9 kg/m^2^, were recruited for the research. Potential participants were excluded if they met any of the following criteria: (1) a history of leg injury, (2) prior back surgery or infection, (3) current back pain, (4) previous experience with hip muscle strength testing, (5) engagement in exercise more than twice a week or participation in competitive sports, (6) currently undergoing treatment for musculoskeletal, systemic, or neurological conditions, (7) pregnant or lactating, or (8) having a BMI exceeding 35 kg/m^2^. The mean age ± standard deviation (SD) of the participants was 21.48 ± 1.50 years (Table 1). Recruitment took place at the Sport Studies Department at the University Putra Malaysia (UPM) in Serdang, Selangor, Malaysia. Written consent was obtained from all participants through an informed consent document. 

### 2.2. Procedures

To ensure accurate data collection, participants in the study were provided with detailed instructions and practice sessions (3 times for each muscle) prior to electrode placement. The skin was carefully prepared by gently abrading it with fine sandpaper and cleansing it with isopropyl alcohol. In some cases, hair was removed by shaving. To capture the EMG data, special dual disposable silver/silver chloride surface recording electrodes were used. The gluteus maximus and medius muscles were studied by means of a 4-channel EMG. These electrodes were strategically positioned within the gluteus maximus and gluteus medius muscles, aligned parallel to the muscle fibers with a precise 20 mm gap between them. Skin impedance was measured using an ohm meter, and readings below 5000 were considered satisfactory [23]. For the gluteus medius muscle, the electrodes were placed above the gluteus maximus muscle, closer to the iliac crest on the lateral side of the pelvis. The electrodes for the gluteus maximus muscle were centered between the sacrum’s posterior superior edge and the higher trochanter’s posterosuperior edge. Finally, a reference electrode was positioned over the anterior superior iliac spine. These electrode placement procedures followed established recommendations from previous researchers, ensuring consistent and reliable data collection methods [21,22].

The study introduced an innovative approach by incorporating an element of unpredictability. The exercises were completed in a random order, keeping participants on their toes and adding excitement to the routine. Each exercise was held isometrically for 5 s, providing a focused challenge to the muscles. To ensure thorough analysis, both the maximum voluntary isometric contraction (MVIC) test and the individual test exercises were repeated three times. Following each repetition, participants were given a brief 30 s rest interval to ensure adequate recovery. Furthermore, a rest period of 5 min, or until the participants felt sufficiently rested to commence the next bout of exercise, was implemented between exercises and repetitions in order to prevent fatigue effects (Table 2). To ensure accuracy, the average of the three readings was used for subsequent analysis, providing a dependable measure of muscle activity. The MVIC test, conducted after completing the test exercises, served as a benchmark to assess the participants’ performance. Verification of electrode placement was crucial and was achieved by examining the EMG signal amplitudes during the manual muscle tests, maintaining consistency with previous studies and upholding methodological integrity [17,21,22].

### 2.3. Statistical Analysis

The researchers employed IBM SPSS software version 22.0 for Windows (SPSS Inc., Chicago, IL, USA) to analyze the collected data. They evaluated the reliability of the EMG recordings for the LM muscle by using the intraclass correlation coefficient (ICC) on the same day that the EMG recordings were obtained. Additionally, they examined the disparities in hip activity between the two exercises through a one-way repeated-measures analysis of variance (ANOVA). To determine statistical significance, the researchers set the significance level at *p* < 0.05. The data are presented in the tables as a mean ± SD percent of MVIC, with a 95% confidence interval (CI). Given the lack of clinically and statistically significant differences between the muscle activity of the two sides during symmetrical exercises, the right and left side data were also averaged so they could be more clearly displayed. The electrodes were placed in a four-square algorithm and smoothed with a 20-millisecond moving window. The amplitude was calculated from a 2 s window centered around the peak activity of the muscles for each of the MVICs and exercises. The maximum EMG signal amplitude during the MVIC of each muscle represented 100% muscle activity. The muscle activity recorded during the exercises was then expressed as a percentage of the MVIC.

## 3. Results

### 3.1. EMG Activity

The summarized findings in Table 3 illustrate the average EMG activity of the gluteus medius and maximus muscles, expressed as a percentage of maximum voluntary isometric contraction (MVIC), for each exercise. The results demonstrated a significant difference in EMG signal amplitude between the single-leg banded glute bridge exercise (mean ± SD: 90 ± 28% MVIC) and the turn-out bent knee pulse exercise (mean ± SD: 70 ± 15% MVIC) (F = 16.584, *p* = 0.001).

The study analyzed the recorded values (mean ± SD percentage of MVIC) from a sample of 64 participants, with a significance level of *p* < 0.05. The results showed that the single-leg banded glute bridge exercise produced significantly higher EMG signal amplitude compared to the turn-out bent knee pulse exercise for both the gluteus medius and gluteus maximus muscles.

### 3.2. Reliability of EMG Recording

The intra-class correlation coefficients (ICCs) and standard errors of the mean (SEM) were determined to assess the reliability of EMG recordings for the gluteus muscles during the turn-out bent knee pulse exercise and the single-leg banded glute bridge exercise on the same day. The ICC value for the turn-out bent knee pulse exercise was 0.95 (SEM, 22.5% MVIC), indicating a high level of consistency in the EMG measurements. Similarly, the ICC value for the single-leg banded glute bridge exercise was 0.83 (SEM, 19.0% MVIC), indicating good reliability in the EMG recordings for this exercise as well.

## 4. Discussion

In this engrossing study, we aimed to compare the activation level of the maximus gluteus and medius gluteus muscles between the turn-out bent knee pulse and the single-leg banded glute bridge exercises: the turn-out bent knee pulse and the single-leg banded glute bridge: the turn-out bent knee pulse and the single-leg banded glute bridge. The goal was to shed light on how these exercises differ and provide valuable insights for physical therapists working with individuals suffering from low back pain.

Significantly, prior investigation has demonstrated a noteworthy correlation between low back pain and the gluteus maximus. Individuals with low back pain were found to experience faster muscle fatigue in their gluteus maximus muscles [16]. This finding has important implications, as avoiding movements that worsen the degenerative spine condition may inadvertently lead to the weakening of the back and hip extensor muscles [17]. Breaking this cycle of pain avoidance behavior is crucial, and recognizing the increased fatigue levels in the gluteus maximus underscores the importance of incorporating this muscle into low back pain rehabilitation [17,18].

But there is more to the story than just pain management. Strengthening the core hip muscles, including the gluteus muscles, can actually boost athletic performance [19]. These muscles play a vital role in generating maximum power and precision during activities like golf and baseball swings [20]. 

This study not only sheds light on the intricate relationship between gluteus muscle activity and low back pain, but it also emphasizes the broader impact of core hip muscle strengthening on both rehabilitation and athletic performance. By exploring these connections, we hope to provide physical therapists with valuable guidance when designing effective treatment plans for patients with low back pain, while also unlocking the secrets to enhanced athletic prowess.

In our study, we observed a noteworthy finding regarding the response of the gluteus medius and gluteus maximus muscles to different exercises. When comparing the turn-out bent knee pulse exercise to the single-leg banded glute bridge exercise, we found that the latter generated significantly higher EMG signal amplitude. When the EMG signal is rectified and smoothed, its amplitude tends to show a positive correlation with the muscle force generation [20]. This suggests that the greater EMG signal amplitude observed during the single-leg banded glute bridge exercise indicates a higher level of muscle activation intensity. Consequently, monitoring the EMG signal amplitude can be a valuable guide for determining exercise intensity when designing exercise programs. It is worth mentioning that previous researchers have reported both linear and nonlinear relationships between EMG signal amplitude and force production during isometric contractions [22]. This further emphasizes the importance of our findings and underscores the potential for utilizing EMG signal amplitudes as a valuable tool in the development of exercise programs.

Physical therapists employ varied approaches in rehabilitating the muscles of the lower back in patients due to the absence of conclusive evidence favoring any specific exercise program [17]. Engaging in the quadruped arm/lower extremity lift exercise revealed a noteworthy observation: one particular muscle, the gluteus maximus, exhibited a remarkable EMG signal amplitude within the strengthening range (mean ± SD: 56% ± 23% MVIC) [21]. In our study, we observed a noteworthy EMG signal amplitude (mean ± SD: 90% ± 28% MVIC) for the gluteus medius and gluteus maximus muscles during the single-leg banded glute bridge exercise, surpassing the values observed during the turn-out bent knee pulse exercise (mean ± SD: 70% ± 15% MVIC). Previous studies have reported a wide range of values (approximately 20% to 40% MVIC) for the gluteus medius and gluteus maximus muscles on the side opposite the lifted lower extremity [24,25].

Researchers have explored various exercises to uncover their impact on the activation of the gluteus medius muscle. Among them, the prone heel squeeze and side-lying hip abduction exercises have shown promising results, regardless of whether they involve internal hip rotation, external hip rotation, or are performed against a wall. These exercises were found to elicit high peak activation levels, reaching around 50% of MVIC [26]. On the other hand, the resisted knee flexion and terminal knee extension exercises yielded rather underwhelming outcomes in terms of muscle activation. These exercises demonstrated minimal levels of activation, hovering around 20% of the MVC [26].

One possibility to consider is that the subjects may have lacked sufficient motivation during the MVIC testing, despite receiving verbal encouragement throughout the process. It is plausible that they did not exert a true maximal contraction during the manual muscle tests [27]. This raises questions about the reliability of the obtained MVIC values.

Interestingly, in our study, we utilized resistance bands to enhance muscle contraction in the gluteus muscles. By addressing these variables, our study offers fresh insights into the activation levels of the gluteus medius and gluteus maximus muscles. This method challenges the existing explanations and sheds light on the potential impact of external support and resistance bands on muscle engagement. These findings contribute to our understanding of optimizing exercise protocols and effectively targeting specific muscle groups for rehabilitation and training purposes.

In an absorbing research conducted by Ekstrom et al. [21], they revealed that the traditional single-leg bridge exercise activates the gluteus maximus at approximately 40% of its MVIC, while the gluteus medius is activated at around 47% of its MVIC [21]. These findings align closely with another study by Lehecka et al. [28], in which they observed remarkable activation levels of 51.01% MVIC for the gluteus maximus and 57.81% MVIC for the gluteus medius while employing the traditional single-leg bridge position. In a captivating study by Youdas et al. [29], they made a noteworthy discovery regarding the activation levels of the gluteus medius muscle during the torso-elevated side support exercise (TESS). Their findings revealed a mean ± SD: 48.1% ± 24.3% MVIC, demonstrating moderate activation. Moreover, our study delved into the single-leg banded glute bridge exercise, which provided an even greater challenge. The results were truly captivating, with a mean ± SD EMG signal amplitude of mean ± SD: 90% ± 28% MVIC for the gluteus medius and mean ± SD: 75% ± 19% MVIC for the gluteus maximus. These findings highlight the exceptional activation levels achieved when engaging in this particular exercise. By pushing the boundaries and exploring more demanding exercises, our study offers valuable insights into maximizing the activation of the gluteus medius and gluteus maximus muscles. These findings can inform the development of targeted rehabilitation and training programs to enhance the strength and functionality of these important muscle groups.

In our study, we sought to address this issue by devising exercises utilizing a resistance band, and we aimed to enable subjects to perform the exercises effectively, even if they had weaker core musculature. This approach allows individuals to gradually build strength and work towards eventually incorporating the challenging exercises identified in previous studies.

By tailoring the exercises to the capabilities of the individuals and providing the necessary assistance, our study offers a valuable alternative for patients in the early stages of their strengthening journey. It highlights the importance of considering individual abilities and implementing exercises that strike the right balance between challenge and attainability.

In Bolgla’s [30] exploration of gluteus medius exercises, a standout performer emerged: the pelvic drop exercise, which demonstrated an impressive activation level of 57% of MVIC [30]. Remarkably, this finding closely aligns with the value of 58% MVIC reported in the study conducted by Boren et al. [31]. The significance of the pelvic drop exercise should not be underestimated. It serves as a functional training exercise specifically targeting pelvic stabilization during the single limb stance. This exercise holds considerable value, as it addresses the crucial role of the gluteus medius muscle in effectively stabilizing the pelvis. Dysfunction in this muscle can lead to a multitude of gait abnormalities and lower extremity pathologies.

Furthermore, in our study, we delved into the single-leg banded glute bridge exercise, which exhibited compelling results in terms of gluteus medius activation. The MVIC measurements from our study reported even higher activity levels, reaching an impressive 90% MVIC for the gluteus medius. This exercise, utilizing a resistance band and focusing on single-leg movement, offers a powerful means of engaging and strengthening the gluteus medius muscle.

These findings highlight the diverse range of exercises available to target and enhance the gluteus medius muscle. By incorporating exercises like the pelvic drop and the single-leg banded glute bridge into training regimens, individuals can improve their pelvic stability, address muscle imbalances, and enhance overall lower extremity function.

In the captivating research conducted by Krause et al. [32], they explored the activation of the gluteus medius muscle during the single-leg stance. Notably, their study utilized EMG measurements; however, they did not introduce any external forces, such as a pulley or cable system. Instead, they relied on body weight resistance, aiming to replicate the exercises commonly prescribed for gluteus medius strengthening. Their investigation took a significant twist, as they sought to enhance the demand on the gluteus medius by manipulating the surface condition. Although the results did not reach a level of statistical significance, there was a noticeable trend indicating increased recruitment of the gluteus medius when performing single limb exercises on an unstable surface. This finding suggests the potential benefits of incorporating instability into training regimens to target the gluteus medius.

In our research protocol, we took a similar approach by incorporating EMG measurements during a single-leg exercise. However, we introduced a notable modification. Instead of relying solely on body weight resistance, we incorporated a banded system, adding additional external force. This modification allowed us to reach a level of statistical significance, surpassing the findings of the Krause study [32]. By utilizing the resistance band, we were able to enhance the demand and engagement of the gluteus medius to a greater extent. This comparison highlights the potential influence of external forces and surface conditions on gluteus medius activation. It offers valuable insights into optimizing exercise protocols for gluteus medius strengthening and underscores the significance of incorporating additional external forces, such as resistance bands, to elicit substantial muscle recruitment.

Notably, studies indicate that achieving EMG amplitudes surpassing the range of 40–60% of the MVIC can provide an adequate stimulus to strengthen muscles [21,22]. With this in mind, the findings from our study unveil the potential of two specific exercises, namely the single-leg banded glute bridge and the turn-out bent knee pulse exercises, to elicit EMG amplitudes that fall within the strengthening range for both the gluteus medius and gluteus maximus muscles.

These results hold great significance, as they imply that incorporating these exercises into rehabilitation protocols may offer valuable benefits for strengthening the gluteus muscles. This newfound understanding may prompt clinicians to consider recommending these exercises as part of their rehabilitation strategies, particularly for individuals experiencing low back pain. Although these exercises can be effective at any stage, they may prove particularly valuable during the initial phases of rehabilitation, helping to improve endurance, stability, and motor control.

By harnessing the power of these targeted exercises, clinicians and individuals alike can strive to enhance the strength and functionality of the gluteus muscles. This newfound knowledge contributes to the growing repertoire of evidence-based approaches in the field of rehabilitation, empowering practitioners to optimize treatment plans and improve patient outcomes.

### 4.1. Limitations of the Study

In our study, it is important to acknowledge several limitations that may impact the generalizability and comprehensiveness of the findings. Firstly, the study sample consisted solely of young females, which restricts the extent to which the results can be extrapolated to other populations, such as males or older individuals. Further research is necessary to ascertain whether similar outcomes can be observed across different demographics, broadening the scope of our understanding.

Secondly, our study focused specifically on comparing the single-leg banded glute bridge and turn-out bent knee pulse exercises. While these exercises were selected for their potential to target the gluteus muscles, it is crucial to recognize that there may be other exercises that were not included in our investigation which are also capable of activating these muscles. As a result, the findings may not fully capture the overall effectiveness of exercises targeting gluteus muscle activation.

Furthermore, it is important to acknowledge the limitations of electromyography (EMG), the method employed to assess muscle activity. Factors such as electrode placement, skin impedance, and cross-talk from adjacent muscles can introduce inaccuracies into EMG measurements, potentially impacting the reliability of the results. Future studies could explore the inclusion of additional methods, such as force plate analysis or motion capture, to provide a more comprehensive evaluation of muscle activation patterns.

Another limitation is that our study focused solely on measuring EMG activity and did not assess functional outcomes or performance improvements resulting from the exercises. To gain a more holistic understanding of their effectiveness, future research should consider evaluating the impact of these exercises on functional tasks or sports-specific movements.

Additionally, it is important to recognize that our study only measured muscle activity during a single exercise session. The long-term effects and the sustainability of the observed differences in muscle activation were not investigated. Future studies could explore the effects of these exercises over an extended period to ascertain their potential for long-term muscle strengthening.

Overall, while our study provides valuable insights into the differential muscle activation between the single-leg banded glute bridge and turn-out bent knee pulse exercises, it is crucial to consider these limitations when interpreting the results and applying them to clinical practice or exercise programs. Further research that addresses these limitations can contribute to a more comprehensive understanding of the topic.

### 4.2. Strengths and Practical Implications of the Study

This study stands out for its robust methodology, employing EMG as an objective measure of muscle activity. The utilization of EMG ensures reliable and precise data collection, providing valuable quantitative insights. Notably, the findings suggest that incorporating the single-leg banded glute bridge exercise into rehabilitation protocols can yield significant benefits for individuals grappling with low back pain or seeking to strengthen their gluteus muscles.

Clinicians can leverage these findings to recommend the single-leg banded glute bridge exercise, as it proves effective in enhancing endurance, stability, and motor control. The study underscores the importance of exercise selection in targeting specific muscles, emphasizing the role of this particular exercise in strengthening the gluteus muscles. The incorporation of external resistance, such as resistance bands, emerges as a key factor in optimizing gluteus muscle activation.

These practical implications hold considerable value for both clinicians and individuals seeking to improve their muscle strength and overall functional outcomes. By incorporating the single-leg banded glute bridge exercise into exercise programs, clinicians can enhance the rehabilitation process and facilitate more favorable patient outcomes. Furthermore, the study’s findings empower individuals to make informed decisions when selecting exercises that maximize muscle engagement and optimize their desired outcomes.

In conclusion, this study’s strengths lie in its rigorous methodology, direct comparison of exercises, and an adequate sample size. The practical implications include incorporating the single-leg banded glute bridge exercise into rehabilitation protocols, guiding exercise selection for gluteus muscle strengthening, and leveraging external resistance to enhance muscle activation. These insights contribute to the advancement of evidence-based practices in rehabilitation and provide a solid foundation for tailored exercise programs targeting the gluteus muscles.

## 5. Conclusions

This study revealed a notable difference in EMG activity between two exercises: the single-leg banded glute bridge exercise and the turn-out bent knee pulse exercise. Remarkably, the single-leg banded glute bridge exercise displayed significantly higher EMG activity in both the gluteus medius and maximus muscles compared to the turn-out bent knee pulse exercise. These findings hold significant promise, suggesting that integrating the single-leg banded glute bridge exercise into rehabilitation protocols may generate valuable benefits in strengthening the gluteus muscles, especially for individuals grappling with low back pain. This newfound understanding has the potential to ignite a shift in clinical practice, prompting healthcare professionals to consider recommending these exercises as a vital component of rehabilitation strategies, particularly during the crucial early stages. By incorporating these exercises, patients can target essential aspects such as endurance, stability, and motor control, which are instrumental in the recovery process.

Harnessing the power of these targeted exercises empowers clinicians and individuals to unlock the true potential of the gluteus muscles, effectively improving their strength and functionality. Ultimately, this novel approach has the power to revolutionize patient outcomes, paving the way for more effective and successful rehabilitation journeys.

## Figures and Tables

**Figure 1:**
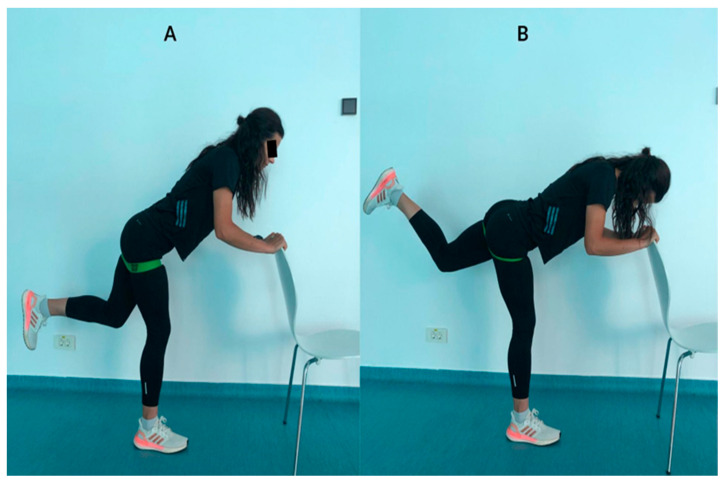
Turn-out bent knee pulse exercise.

**Figure 2:**
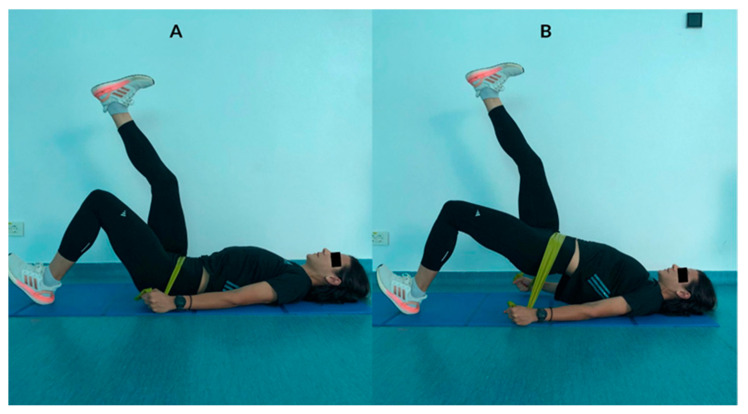
Single-leg banded glute bridge exercise.

**Table 1:** Baseline data of subjects (*n* = 64).

	Mean	SD
Age (years)	21.48	1.50
Height (cm)	152.86	13.7
Body mass (kg)	50.65	7.43
BMI (kg/m^2^)	20.47	1.31

**Table 2:** The methods employed for performing the chosen exercises.

Exercise	Description
Turn-Out Bent Knee Pulse Exercise (Figure 1)	Assume the starting position by gripping the top of a chair for support while having a resistance band positioned below your pelvis. Your left leg should be positioned somewhat close to the ground. Now, gradually raise your left leg as high as you can and hold it in that elevated position for a count of 5 s. Return to the initial starting position and repeat the exercise. This exercise aims to strengthen and improve the flexibility of your gluteus muscles while incorporating resistance training for added challenge.
Single-Leg Banded Glute Bridge Exercise (Figure 2)	Introducing a subtle variation to the traditional single-leg bridge exercise, this is a modified version that adds an interesting twist. Assume the starting position with your left heel firmly planted on the ground, while extending your right foot upwards. Take hold of the resistance band with both hands, positioning it across your hips. Now, utilize the strength of your right heel to drive your hips upward, creating a bridge-like shape with your body, and hold this position for a count of 5 s. Return to the initial starting position and repeat the exercise. This modified single-leg bridge not only targets and strengthens your gluteus muscles, but also engages your core and adds an element of resistance training for an even more effective workout.

**Table 3:** EMG activity of the gluteus muscles during the turn-out bent knee pulse exercise and the single-leg banded glute bridge exercise (*n* = 64) (EMG activity (%MVIC)).

Exercise	Gluteus Medius Test (Mean ± SD)	Gluteus Medius Retest (Mean ± SD)	Gluteus Maximus Test (Mean ± SD)	Gluteus Maximus Test (Mean ± SD)	Gluteus Maximus Retest (Mean ± SD)	ICC	95% CI for ICC
Turn-Out Bent Knee Pulse Exercise	70 ± 15	71 ± 10	60 ± 20	60 ± 20	59 ± 13	0.83	0.83–0.85
Single-Leg Banded Glute Bridge Exercise	90 ± 28	86 ± 30	75 ± 19	75 ± 19	74 ± 20	0.95	0.91–0.96

## Data Availability

For those interested, the data from this study can be obtained by contacting the corresponding author. However, please note that the data is not publicly accessible due to privacy limitations.
